# Removal of Pulps Painlessly

**Published:** 1903-07-15

**Authors:** W. C. Davis


### REMOVAL OF PULPS PAINLESSLY.

By this method the pulp can be removed without pain from any tooth under any circumstances, even in the case of a child, in from one to three minutes. The method is as follows: Apply the dam if possible and dry the cavity. If the pulp is not exposed but covered with a layer of softened dentine, apply first a drop of adrenalin chloride solution, then one drop of a forty percent solution of formaldehyde. If quite a distance from the pulp, use slight but continued pressure with a rubber plug for a few seconds. It will now be possible to painlessly excavate to complete or nearly complete exposure.

The author's process of devitalization may be said to consist of three steps, which he thus describes:

1st. Apply to the cavity one drop of adrenalin chloride solution, 1-1000 (best applied by taking some up in the operating pliers between the points of which it will be retained by capillary attraction).

2nd. Place in the cavity a few crystals of cocaine or a small $\frac{1}{6}$-grain soluble tablet of the drug.

3rd. Apply one drop of a forty-percent solution of formaldehyde.

4th. Apply pressure with a rubber plug, at first very lightly, though steadily, but not so firmly as to cause the patient any pain. Gradually increase the pressure until at the end of forty to sixty seconds the rubber can be kneaded into the cavity with the amalgam burnisher with the necessary force which should cause the patient no pain. Now remove the covering of the pulp chamber and pass a broach slowly toward the apex. If there is a tendency to much hemorrhage, or the patient should feel to the slightest degree the movements of the broach, repeat the method again, being sure not to omit the formaldehyde, when you can remove the pulp of any tooth without the patient feeling the operation. In cases in which the tooth is slightly sore to percussion, as in the advanced stage of pulpitis, the soreness will have disappeared. Many times I find that the peridental membrane has lost its tactile sense. In cases in which the apical foramen is large and hemorrhage recurs, apply the adrenalin only, with pressure, for fifteen seconds, after which not another drop of blood will be discharged. Those who prefer can now proceed with root-filling. I prefer to place in the root a non-irritant dressing (mine is campho-phenique), which remains for twenty-four hours, and then to fill.

The advantages of the above method are:

1st, it is painless; 2nd, it saves time; 3rd, the color of the tooth is never changed; 4th, the after-soreness is slight, and many times wanting; 5th, the application is a powerful antiseptic.

The author insists that success by this method is assured, provided the various steps be followed in the exact order in which he has enumerated them. The adrenalin chloride sotution must be applied first to contract the pulp and draw it away from the exposure to prevent pain.—W. C. Davis, in *Items of Interest.*

